# Supplementary material for: Resolving the full spectrum of human genome variation using Linked-Reads
Source: Genome Res. 2019 Apr;29(4):635–45. doi: 10.1101/gr.234443.118 (PMC6442396; doi:10.1101/gr.234443.118)
Supplement: Supplemental Material [file supp_gr.234443.118_Supplemental_longranger-2.2.zip › longranger-2.2.tar.bz2!/lr2.2-public/lib/stan/cmdstan/stan/doc/api/html/agrad_2fwd_2matrix_2rows__dot__product_8hpp.html]

Stan: src/stan/agrad/fwd/matrix/rows\_dot\_product.hpp File Reference


|  |  |
| --- | --- |
|  | Stan  2.5.0  probability, sampling & optimization |


- Main Page
- Related Pages
- Namespaces
- Classes
- Files

- File List
- File Members

All Classes Namespaces Files Functions Variables Typedefs Enumerations Enumerator Friends Macros Pages

- src
- stan
- agrad
- fwd
- matrix

Namespaces |
Functions

rows\_dot\_product.hpp File Reference

`#include <vector>`  
`#include <stan/math/matrix/Eigen.hpp>`  
`#include <stan/math/matrix/typedefs.hpp>`  
`#include <stan/math/error_handling/matrix/check_matching_dims.hpp>`  
`#include <stan/agrad/fwd/matrix/typedefs.hpp>`  
`#include <stan/agrad/fwd/matrix/dot_product.hpp>`  
`#include <stan/agrad/fwd/fvar.hpp>`

Go to the source code of this file.

|  |  |
| --- | --- |
| Namespaces | |
|  | stan |
|  | Probability, optimization and sampling library. |
|  | |
|  | stan::agrad |
|  | Function gradients via reverse-mode automatic differentiation. |
|  | |

|  |  |
| --- | --- |
| Functions | |
| template<typename T , int R1, int C1, int R2, int C2> | |
| Eigen::Matrix< fvar< T >, R1, 1 > | stan::agrad::rows\_dot\_product (const Eigen::Matrix< fvar< T >, R1, C1 > &v1, const Eigen::Matrix< fvar< T >, R2, C2 > &v2) |
|  | |
| template<typename T , int R1, int C1, int R2, int C2> | |
| Eigen::Matrix< fvar< T >, R1, 1 > | stan::agrad::rows\_dot\_product (const Eigen::Matrix< double, R1, C1 > &v1, const Eigen::Matrix< fvar< T >, R2, C2 > &v2) |
|  | |
| template<typename T , int R1, int C1, int R2, int C2> | |
| Eigen::Matrix< fvar< T >, R1, 1 > | stan::agrad::rows\_dot\_product (const Eigen::Matrix< fvar< T >, R1, C1 > &v1, const Eigen::Matrix< double, R2, C2 > &v2) |
|  | |


---

[ Stan Home Page ]

*© 2011–2014,
Stan Development Team.*
